# Numbers Game and Immune Geography as Determinants of Coronavirus Pathogenicity

**DOI:** 10.3389/fcimb.2020.559209

**Published:** 2020-10-23

**Authors:** Gennady Bocharov, Valentina Casella, Jordi Argilaguet, Dmitry Grebennikov, Roberto Güerri-Fernandez, Burkhard Ludewig, Andreas Meyerhans

**Affiliations:** ^1^Marchuk Institute of Numerical Mathematics, Russian Academy of Sciences, Moscow, Russia; ^2^Moscow Center for Fundamental and Applied Mathematics at Marchuk Institute of Numerical Mathematics, Russian Academy of Sciences (INM RAS), Moscow, Russia; ^3^Institute for Personalized Medicine, Sechenov First Moscow State Medical University, Moscow, Russia; ^4^Infection Biology Laboratory, Department of Experimental and Health Sciences, Universitat Pompeu Fabra, Barcelona, Spain; ^5^Institut de Recerca i Tecnologia Agroalimentàries (IRTA), Centre de Recerca en Sanitat Animal (CReSA, IRTA-UAB), Campus de la Universitat Autònoma de Barcelona, Bellaterra, Spain; ^6^Infectious Diseases Unit, Hospital del Mar-IMIM, Universitat Autònoma de Barcelona, Barcelona, Spain; ^7^Institute for Immunobiology, Kantonsspital St.Gallen, St. Gallen, Switzerland; ^8^Institució Catalana de Recerca i Estudis Avançats (ICREA), Barcelona, Spain

**Keywords:** COVID-19, SARS - CoV-2, IFN-I, innate immunity, viral dynamics, pathogenesis

Once again, the new SARS-CoV-2 reminds us about the potential of infectious pathogens to fiercely spread worldwide and puts our well-being in danger. With respect to its pathogenicity, one may be reminded of two simple principles that underlie our constant fight with viruses of our surrounding, the “numbers game” and “immune geography” (Zinkernagel et al., 1985). These conceptual terms refer to the kinetics of virus growth and immune reactions, and the spatial organization of immune responses in relation to viral entry sites and tissue tropism which determines cytokine gradients and immune cell homing patterns across tissues, respectively. Jointly, both concepts provide a rationale for a dynamic view on the balance between virus expansion and development of antiviral immune responses that will ultimately help to understand virus pathogenicity and how to gain immune control (Figure 1A).

**Figure 1:**
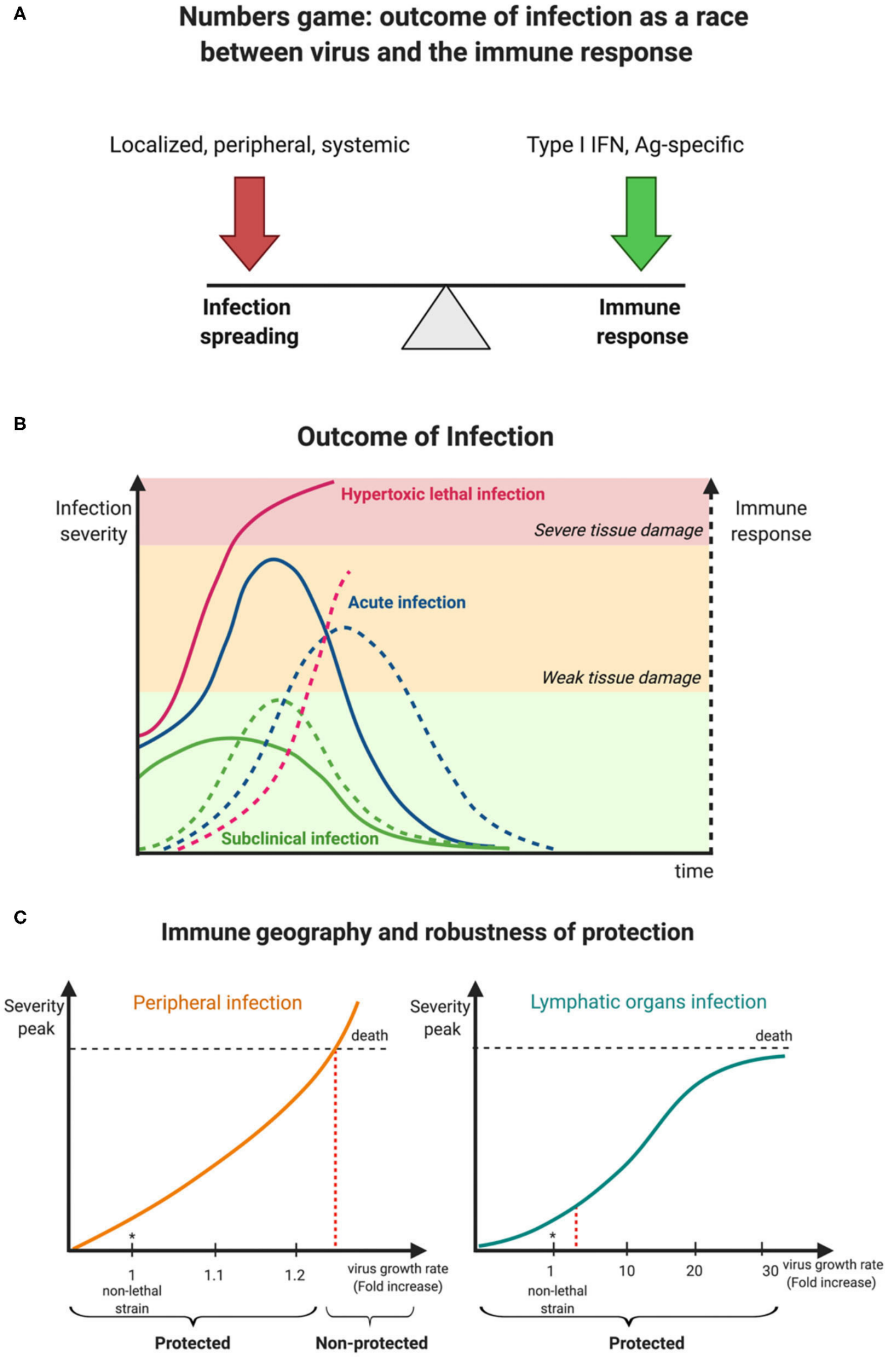
Kinetic view on CoV pathogenesis. **(A)** The numbers game between virus growth and immune response determines virus infection outcomes. **(B)** Early innate immune responses determine viral pathogenesis. **(C)** Degree of robustness of protection against a faster replicating M-CoV strain in relation to its preferential site of replication, i.e., a peripheral organ (liver) vs. a lymphoid organ (spleen). Virus is represented as a solid line while broken lines characterize the immune response.

Coronaviruses are a family of enveloped plus-strand RNA viruses that infect mammals and birds. In humans, there are four endemic, usually mildly pathogenic viruses 229E, OC43, NL63 and HKU1, and the newly emerged viruses SARS-CoV, MERS-CoV, and SARS-CoV-2 that cause lethal infections in around 10, 30, and 1% of cases, respectively (Gaunt et al., 2010; Raoult et al., 2020). All these viruses can be transmitted by aerosols and infect the upper respiratory tract and the lung. However, since human infections only allow for very limited experimental manipulations, much of our fundamental knowledge of virus infections are derived from animal studies. To understand the containment of the initial virus spread, which subsequently determines virus pathogenicity, the murine coronavirus (M-CoV, also known as mouse hepatitis virus, MHV) model system is most instructive. M-CoV is a rapidly replicating, highly cytopathic virus that leads to severe inflammation in several organs and can disseminate via the bloodstream. In particular, the liver, a peripheral organ and the spleen, a secondary lymphoid organ, are the major target organs of this virus, and hematopoietic cell-derived type I interferon (IFN) primarily controls viral replication and virus-induced liver disease. Three clinical outcomes are observable that depend on the balance of infection spreading and the innate immune response (Figure 1B). With an optimal innate response, the infection is well controlled and generates only mild symptoms, whereas, a delayed and weak innate response results in severe infection spread and fatal outcome. Between these extremes, tissue damage of various severity is observed but the host ultimately survives.

How can this qualitative description of virus-induced pathogenicity be transformed into a more quantitative characterization of host protection and its limits? A systems biology-based analysis combining experimental data of murine coronavirus infection and mathematical modeling has shown that the robustness of innate immune response-mediated protection from severe disease is rather limited (summarized in Figure 1C) when putative CoV variants exhibiting enhanced tropism and replication in peripheral organ cells like hepatocytes rather than lymphoid tissue cells were considered (Bocharov et al., 2010, 2018). For example, a 20% increase of the exponential growth rate of M-CoV A59 strain (estimated to be 0.78 infectious particles/ml/h) leads to a severe infection as manifested by a substantial rise of liver enzyme values in serum (i.e., alanine transferase (ALT) level rise to 1,000 IU/L) within 2 days. On the contrary, a substantial rise of up to 30-fold of the virus replication rate in macrophages of the spleen (estimated to be about 37 infectious particles/cell/h for M-CoV A59 in C57BL/6 mice) is efficiently disarmed by plasmacytoid dendritic cell-produced type I IFN, thus providing robust protection against severe disease (Bocharov et al., 2010). In general terms, differences of innate immune responses in lymphoid tissues such as the spleen or lymph nodes in which local levels of type I IFN increase sharply when viruses replicate, provide a swift host protection, whereas virus replication in non-lymphoid tissue like liver or lung generate slower type I IFN increases reaching lower levels and thus being less efficient in restriction of viral replication.

How do the above numbers relate to the rate of infection spreading in the case of SARS-CoVs? The available experimental data on the virus growth kinetics in Vero E6 cells *in vitro* provide estimates of the exponential growth rates of SARS-CoV-1 to be about 0.74 RNA/ml/h (Keyaerts et al., 2005) and 0.84 infectious particles/ml/h. Recent data for SARS-CoV-2 provide a similar growth rate (Lokugamage et al., 2020). The remarkable similarity of the values and the preferential tropism of the SARS-CoV viruses to cells of the upper respiratory tract and lungs would suggest that the robustness of protection against SARS-CoV spreading in this peripheral organ by the innate IFN response is limited. Thus, it is conceivable that SARS-CoV infections are operating close to the edge of the protective capacity of the host's type I IFN system. Consequently, changes in type I IFN-sensitivity of the viruses, their IFN-suppressive properties as well as changes in type I IFN-responsiveness of infected hosts i.e., due to genetic polymorphisms, immunosuppressive drugs or aging might all have relevant clinical consequences. Indeed, it has already been speculated for SARS- and MERS-CoVs that a delay in virus restriction by the early type I IFN response may lead to exaggerated inflammatory, life-threatening conditions later during the infection course (Prompetchara et al., 2020). With respect to SARS-CoV-2 infections, recent data suggest that an impaired systemic type I IFN response correlates with the severity of COVID-19 (Arunachalam et al., 2020). Furthermore, since aging affects multiple layers of the immune control of viruses including an altered innate response and correlates to COVID-19 severity (Akbar and Gilroy, 2020)(https://ebrary.net/30820/health/pdcs), further longitudinal studies in humans of this complex relationship are warranted.

Clearly, the degree of fragility, i.e., the sensitivity of CoV-induced infection severity to viral infection dose and growth rate increases in humans, and the limits of protection provided by the type I IFN system that acts at the front line of the virus-host interaction deserve further quantitative analyses. The higher type I IFN sensitivity of SARS-CoV-2 compared to SARS-CoV indicates that a type I IFN treatment of a virus-exposed individual would be an efficient early therapeutic modality that could prevent subsequent severe disease. This view is now supported by latest clinical data that show favorable clinical responses and reduced mortality after early type I IFN administration (Wang et al., 2020). Furthermore, given the large reservoir of CoVs in bats (Menachery et al., 2015), testing their properties with respect to growth rates, IFN-sensitivity and IFN-suppressive capacity might help to be prepared for the next pandemic to come. It is only a question of time.

## Author Contributions

VC, JA, DG, RG-F, BL, GB, and AM jointly discussed CoV pathogenesis. GB and AM drafted the manuscript. VC, JA, DG, RG-F, BL, GB, and AM revised the manuscript. All authors contributed to the article and approved the submitted version.

## Conflict of Interest

The authors declare that the research was conducted in the absence of any commercial or financial relationships that could be construed as a potential conflict of interest.
